# The Effect of a Synthetic Estrogen, Ethinylestradiol, on the hERG Block by E-4031

**DOI:** 10.3390/biom11091385

**Published:** 2021-09-20

**Authors:** Fumiya Tamura, Shintaro Sugimoto, Mana Sugimoto, Kazuho Sakamoto, Masahiko Yamaguchi, Takeshi Suzuki, Keiichi Fukuda, Masaki Ieda, Junko Kurokawa

**Affiliations:** 1 Department of Cardiology, Keio University School of Medicine, 35 Shinanomachi, Shinjuku-ku, Tokyo 160-8582, Japan; fumiya.tamura@keio.jp (F.T.); kfukuda@a2.keio.jp (K.F.); 2 Department of Bio-Informational Pharmacology, School of Pharmaceutical Sciences, University of Shizuoka, 52-1 Yada, Suruga-ku, Shizuoka-shi, Shizuoka 422-8526, Japan; s21808@u-shizuoka-ken.ac.jp (S.S.); m17062@u-shizuoka-ken.ac.jp (M.S.); kazuho@u-shizuoka-ken.ac.jp (K.S.); masahiko-y@u-shizuoka-ken.ac.jp (M.Y.); 3 Division of Basic Biological Sciences, Faculty of Pharmacy, Keio University, 1-5-30 Shibakoen, Minato-ku, Tokyo 105-8512, Japan; suzuki-tk@pha.keio.ac.jp; 4 Department of Cardiology, Faculty of Medicine, University of Tsukuba, 1-1-1 Tennoudai, Tsukuba City, Ibaraki 305-8575, Japan; mieda@md.tsukuba.ac.jp

**Keywords:** cardiac potassium channel, hERG blocker, synthetic estrogen, QT intervals, drug interaction

## Abstract

Inhibition of K^+^-conductance through the human ether-a-go-go related gene (hERG) channel leads to QT prolongation and is associated with cardiac arrhythmias. We previously reported that physiological concentrations of some estrogens partially suppress the hERG channel currents by interacting with the S6 residue F656 and increase the sensitivity of hERG blockade by E-4031. Although these studies suggested that clinically used synthetic estrogens with similar structures have the marked potential to alter hERG functions, the hERG interactions with synthetic estrogens have not been assessed. We therefore examined whether ethinylestradiol (EE2), a synthetic estrogen used in oral contraceptives, affects hERG function and blockade by drugs. Supratherapeutic concentrations of EE2 did not alter amplitudes or kinetics of the hERG currents elicited by train pulses at 20 mV (0.1 Hz). On the other hand, EE2 at therapeutic concentrations reduced the degree of hERG current suppression by E-4031. The administration of EE2 followed by E-4031 blockade reversed the current suppression, suggesting that the interaction of EE2 and E-4031 alters hERG at the drug-binding site. The effects of EE2 on hERG blockade raised the possibility that other estrogens, including synthetic estrogens, can alter hERG blockade by drugs that cause QT prolongation and ventricular arrhythmias.

## 1. Introduction

The rapid component of the delayed rectifier potassium current (I_Kr_) plays an essential role in cardiac repolarization [1]. The pore-forming subunit of the human I_Kr_ channel is encoded by the human ether-a-go-go-related gene (hERG; KCNH2) [2]. A decrease in I_Kr_ as a result of drug blockade is a major cause of acquired (drug-induced) long QT syndrome (LQTS), which is associated with electrocardiographical QT_C_ prolongation and lethal ventricular arrhythmias, presenting as torsades de pointes (TdP) [3]. Thus, the hERG channel has become a primary anti-target in drug development. However, drug-induced QT_C_ prolongation is a complicated phenomenon that is related not just to unintended hERG blockade, but also to multi-channel blockade, drug–drug interactions, and a variety of patient factors, including sex [4,5,6,7].

Women are more prone to developing TdP in response to QT-prolonging drugs than men [4,5,6,7], and the mechanism may be related to baseline QT_C_ intervals, which are approximately 20 ms longer in women than in men [7]. The mechanism of sex differences in baseline QT_C_ intervals involves the shortening of QT_C_ intervals, mainly by endogenous testosterone and progesterone [8,9,10,11]. Although the effects of estrogen on QT intervals may not be as dominant as those of testosterone or progesterone, studies of menopausal hormone therapy (MHT) in the form of estrogen-alone therapy (ET) and estrogen plus progesterone therapy (EPT) suggested a counterbalancing effect of exogenous estrogen and progesterone on QT intervals [12]. Specifically, ET lengthens the QT interval, whereas EPT has no effect. In our animal studies [13,14], estrogen at a physiological concentration lengthened QT intervals by suppressing I_Kr_ in a receptor-independent manner [13]. Our previous patch-clamp analysis with hERG channel-expressing cells revealed that some estrogens, estradiol and estrone sulfate, interact with the hERG channel, and alter the effects of a selective hERG blocker, E-4031 [13,15]. This study is consistent with the clinical QT prolongation by estrogens used for ET. Recently, oral contraceptives were reported to increase the risk of TdP based on the administration of *d,l*-sotalol to healthy female volunteers [16]. However, to date, no studies have assessed whether oral contraceptive ingredients affect the hERG channel function. Thus, we investigated the effects of ethinylestradiol (EE2), which is used in almost all modern formulations of combined oral contraceptive pills, on the hERG channel currents and hERG blockade by E-4031 in stable hERG-expressing HEK293 cells.

## 2. Materials and Methods

### 2.1. Materials

Stock solutions of EE2 (Tokyo Chemical Industry Co. Ltd., Tokyo, Japan) at 10 mM (in ethanol) and E-4031 (Eisai Co. Ltd., Tokyo, Japan) at 10 mM (in H_2_O) were diluted to final concentrations in the external solutions. The final concentration of solvent (ethanol) was confirmed to have no effects on hERG currents [13]. All other materials were of reagent grade quality and obtained from standard sources.

### 2.2. Cell Culture

Human embryonic kidney (HEK) 293 cells stably expressing hERG [17] were cultured in phenol red-free D-MEM supplemented with 10% charcoal-treated FBS and 200 µg/mL of geneticin, G418. All cells were kept in an incubator at 37 °C with 5% CO_2_, and plated on culture dishes the day before electrophysiological experiments.

### 2.3. Electrophysiology

Methods were described in detail previously [13]. In brief, hERG channel currents were recorded at room temperature (22 ± 2 °C) using the perforated patch-clamp technique with an Axopatch 200B amplifier (Molecular Devices, San Jose, CA, USA). The control bath solution contained 132 mM NaCl, 4.8 mM KCl, 1.2 mM MgCl_2_, 2 mM CaCl_2_, 5 mM glucose, and 10 mM HEPES, pH 7.4. Pipettes (2–4 MΩ resistance) were filled with a solution containing 110 mM K-aspartate, 5 mM ATP-K_2_, 1 mM CaCl_2_, 1 mM MgCl_2_, 11 mM EGTA, and 5 mM HEPES, pH 7.3. To achieve patch perforation (series resistance: 10–20 MΩ), amphotericin B (0.3 mg/mL) (Nacalai Tesque, Inc., Kyoto, Japan) was added to the pipette solution. Signals were low-pass filtered at 5 kHz, sampled at 2 kHz, and compensated for cell capacitance (10–40 pF), but not for series resistance.

To investigate the effects of EE2 on hERG current amplitudes, the peak deactivating tail current was recorded at the repolarizing steps to −40 mV subsequent to 2-s depolarizing test pulses to 20 mV from a holding potential of −80 mV, as described previously [13]. The hERG channel tail-current amplitude was monitored at 0.1 Hz. In order to examine the hERG activation curves, the data of the normalized tail current amplitudes of I_hERG_ were fitted to the Boltzmann equation: I/I_max_ = G/G_max_ = {1 + exp [−(V_m_ − V_0.5_)/*k*] −1}, where G/G_max_ is normalized chord conductance at V_m_ to the maximum chord conductance, V_0.5_ is the potential where the conductance is half-maximally activated, and *k* is the slope factor.

### 2.4. Data Analysis

**Figure 1:**
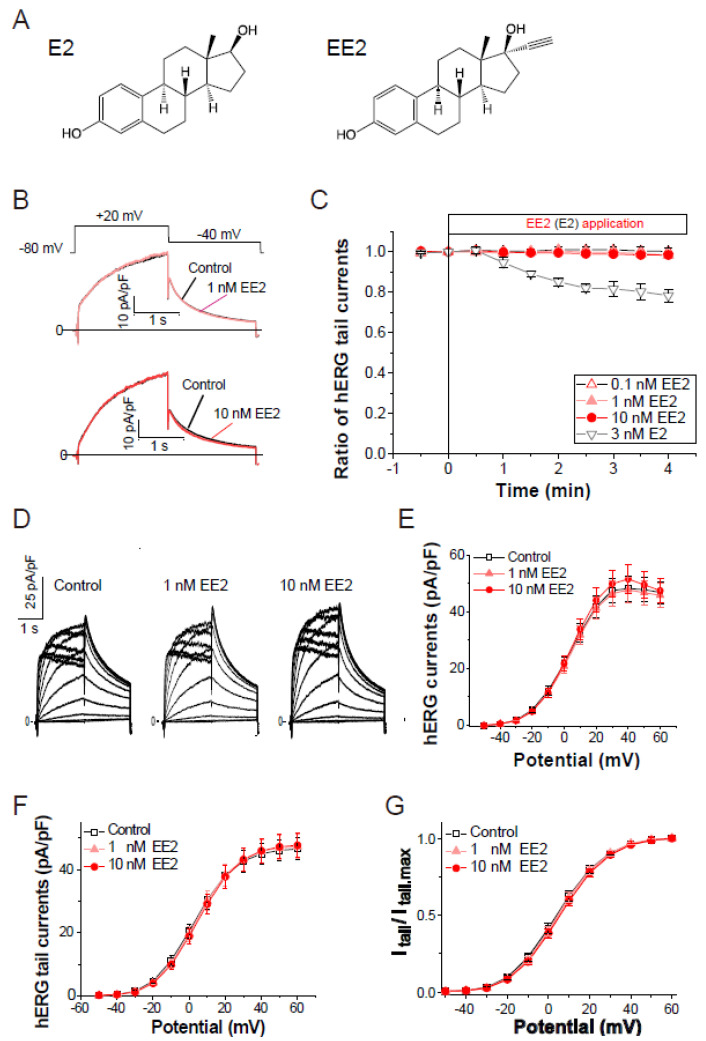
Effects of the external application of ethinylestradiol, EE2, on hERG channel currents. Membrane currents were recorded from HEK293 cells stably expressing hERG. The hERG channels were sequentially activated at 0.1 Hz by 2-s test pulses from a holding potential (V_h_) at −80 mV. (**A**), Chemical structures of E2 (left) and EE2 (right). (**B**), Representative traces before and after the application of EE2 (upper; 1 nM, lower; 10 nM) for 4 min. (**C**), Time courses of the effects of EE2 at 0.1 nM (open triangles, *n* = 5), 1 nM (closed triangles, *n* = 8), and 10 nM (*n* = 7), and E2 at 3 nM (closed triangles, *n* = 5). After stabilizing the tail amplitudes for 1 min, currents were recorded in the presence of each concentration of EE2 in the bath solution. Plots (means ± S.E.M.) are shown as ratios of the peak tail amplitudes just before the application of estrogens (control at time zero). (**D**–**G**), No effect on current-voltage relationships of the hERG channel elicited by a series of 2-s test pulses from −50 to 60 mV (10-mV increments, 0.1 Hz). (**D**), Representative superimposed traces at step pulses (2-s test pulses, −40 mV return, V_h_ = −80 mV) from a single cell before (left) and after a 5-min cumulative application of EE2 from 1 nM (middle) to 10 nM (right). (**E**), Current–voltage relationship at the end of test pulses. (**F**), Current–voltage relationship of tail peak currents recorded at −40 mV. (**G**), Normalized peaks of tail currents were plotted as a function of the hERG activation (Boltzmann fitting). Eighteen cells were used. Data are summarized in Table 1.

**Figure 2:**
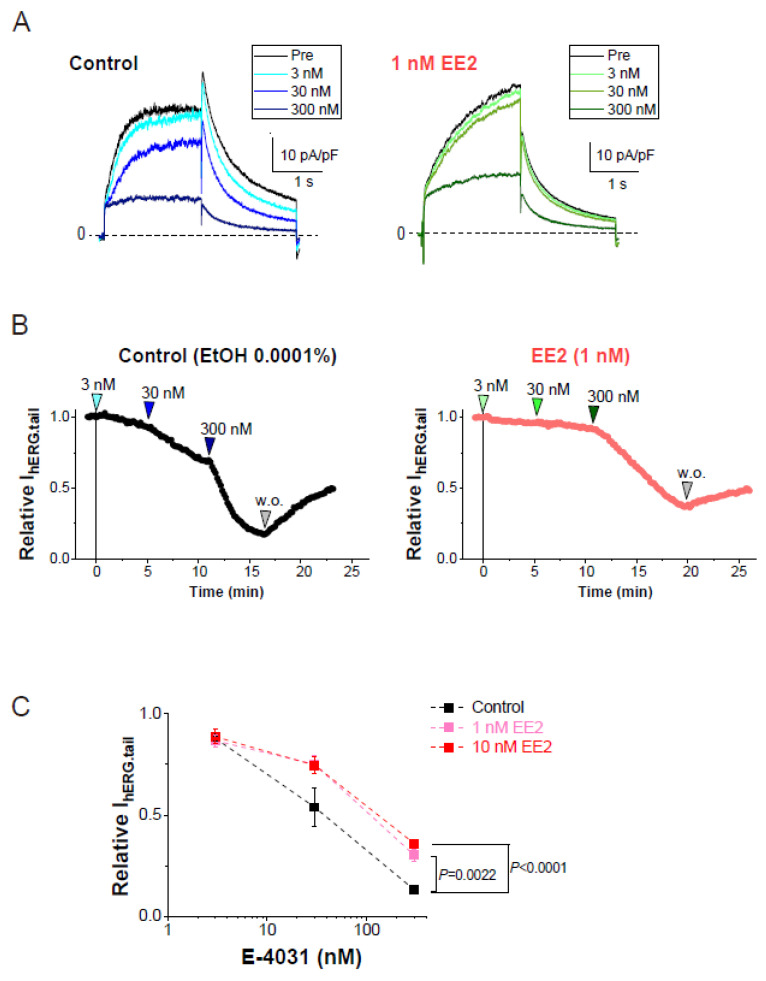
Blockade of hERG currents by E-4031 with or without EE2. hERG currents were recorded from HEK293 cells using the scheme as that described in Figure 1D. The hERG channels were sequentially activated at 0.1 Hz by 20-mV test pulses for 2 s from a holding potential (V_h_) at −80 mV, whereas tail currents were recorded with a repolarizing step to −40 mV. EE2 at 1 nM was administered 5 min prior to the cumulative application of E-4031. (**A**). Representative traces in the presence of EtOH (left) and EE2 (right) are shown by superimposing the traces before (control) and after the addition of E-4031 (3, 30 and 300 nM). Scale, 10 pA/pF, 1 s. (**B**). Time course of hERG inhibition by cumulative application of E-4031 in the presence of 0.0001% EtOH (left) and 1 nM EE2 (right). After the current amplitudes were stabilized for 1 min, E-4031 was added in the presence of EtOH or EE2. The timing of E-4031 applications (3, 30, and 300 nM) indicated by the arrowheads above the plot. (**C**). Concentration-dependent inhibition of E-4031 was plotted as relative values of the tail amplitudes compared to the right before the application of E-4031. Colored lines indicate the presence of EE2. Control (no EE2); *n* = 8, EE2 at 1 nM; *n* = 8, EE2 at 10 nM; *n* = 8.

All values are presented as the mean ±S.E. pCLAMP 10.7 software (Molecular Devices, San Jose, CA, USA) was used to both acquire and analyze data for the patch-clamp experiments. Graphical and statistical analyses were carried out using OriginPro 2021 software (OriginLab Corporation, Northampton, MA, USA). Significant differences for multiple comparisons in Figures 1C and 2 were assessed using one-way ANOVA followed by Tukey’s post hoc test. Statistical analysis was carried out using SPSS software Ver27.0 (IBM, Armonk, NY, USA). *p* < 0.05 was considered to be significant.

## 3. Results

To assess the effects of ethinylestradiol (EE2, Figure 1A) on the hERG current, patch clamp analysis in stable hERG-HEK cells was performed as described in the Methods. The hERG channel function was measured as the peak tail current at −40 mV following a 2-s voltage step from −80 mV to the test voltages (V_t_). As shown in Figure 1B,C, exposure of EE2 (0.1–10 nM) had no effect on hERG channel current traces elicited by a test pulse at 20 mV. Under the same experimental condition, exposure to 3 nM 17-β-estradiol (E2, Figure 1A,C) for 4 min significantly blocked hERG tail currents by 21.6 ± 3.2% (*p* < 0.00001 ANOVA with repeated measures, vs. EE2), confirming the acute and partial hERG block by E2 [13]. The effects of EE2 on current–voltage (I–V) relationships for peak outward hERG currents and peak tail currents were investigated by applying EE2 at 1 nM and 10 nM, respectively (Figure 1D–F). For each cell, the respective tail peak amplitudes at each step were normalized to the amplitudes of maximum tail current elicited by a strong V_t_ to the plateau level of channel activation, allowing us to estimate the macroscopic channel availability at the end of the preceding V_t_. As shown in Table 1, which summarizes the data analysis, the 5-min application of EE2 did not affect the maximum hERG current density or voltage dependence of the activation. These analyses for voltage-dependent activation of the hERG channel revealed that EE2 at clinical dosages has no direct effects on the hERG channel activity.

We next evaluated the effects of EE2 on the sensitivity of a hERG blocker, E-4031, to hERG currents in HEK293 cells. In the presence or absence of hormones, plots of tail amplitudes were normalized relative to the values just before the application of E-4031. Experiments in the presence or absence of hormones were performed on the same day to avoid the effects of unexpected batch-to-batch variation between cell cultures. As shown in Figure 2, the presence of EE2 in the external solution significantly reduced the fractional inhibition of hERG currents induced by E-4031 at 300 nM.

**Figure 3:**
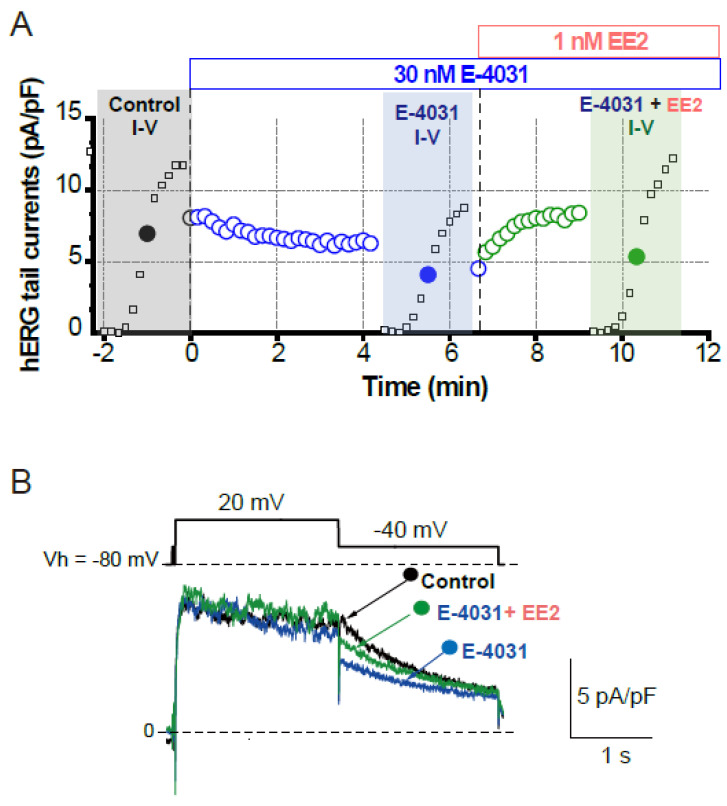
Effects of EE2 on blockade of hERG currents by E-4031. HERG currents were recorded from HEK293 cells stably expressing hERG as described in Figure 1. (**A**). Time course of hERG tail current blockade by E-4031 at 30 nM and the following recovery by the addition of EE2 at 1 nM. Plots from a representative experiment are normalized by the tail amplitude before E-4031 application (time 0). The current-voltage (I–V) relationships were tested before drug application (control, black), after E-4031 block (blue) and after the addition of EE2 (E-4031 + EE2, green). (**B**). Representative traces elicited by 20-mV test pulse (closed circles in I–V relationships) are shown by superimposing the traces before (control, black) and after the application of E-4031 only (blue) and E-4031 plus EE2 (green). Scale, 5 pA/pF, 1 s.

**Figure 4:**
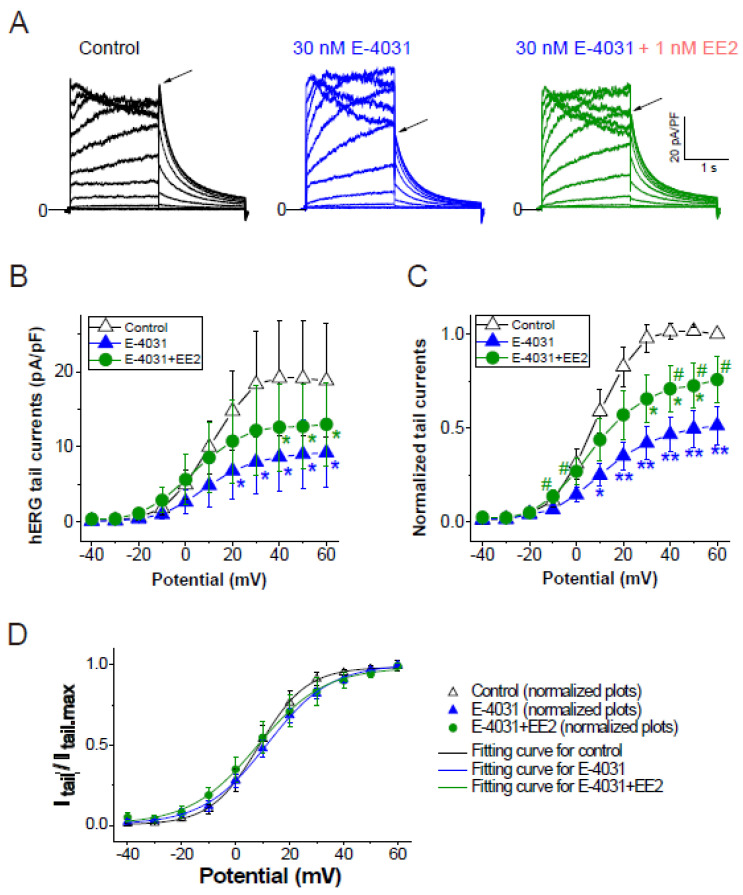
Effects of EE2 on I–V relationships of hERG currents by E-4031. I–V relationships of the hERG channel were activated by the same voltage protocol described in Figure 1D, and compared before drug application (control, black), after 30 nM E-4031 block (blue), and after the addition of EE2 (30 nM E-4031 + 1 nM EE2, green). (**A**), Representative traces elicited by step pulses (−40 mV to 60 mV). Tail peaks are indicated by arrows. Scale, 20 pA/pF, 1 s. (**B**), Tail I-V curves before (control), after the application of E-4031, and after the addition of EE2 (E-4031 + EE2). *p* < 0.05 ANOVA with repeated measures. * *p* < 0.05 vs. control. (**C**), Respective tail peak amplitudes were normalized to the tail amplitude preceding 60 mV before drug applications (control). *p* < 0.05 ANOVA with repeated measures. * *p* < 0.05, ** *p* < 0.01, vs. control, # *p* < 0.05 vs. E-4031. (**D**), Comparison of channel availability curves obtained from tail I-Vs. Smooth lines are Boltzmann fits, as described in the Methods, that generated V0.5 of activation in Control (black line), in E-4031 (blue line), and in E-4031/EE2 (green line); ns, ANOVA. Seven experiments were performed. Significance was evaluated using Student’s t-tests after repeated measures one-way ANOVA.

To assess whether later-administered EE2 can alter the hERG blockade by E-4031, EE2 at 1 nM was added after the hERG blockade was stabilized by E-4031 at 30 nM (Figure 3). A time course of the peak tail hERG currents at −40 mV is depicted in Figure 3A. To investigate the effects of EE2 on the hERG channel blockade by E-4031, the peak tail hERG amplitudes were compared under each administrative condition (Figure 3 and Figure 4). E-4031 at 30 nM near IC_50_ [13] inhibited the peak tail currents of the hERG channel (Figure 3A, blue plots), and the following addition of EE2 at 1 nM restored the tail amplitudes in a time-dependent manner (Figure 3A, green plots). To examine the voltage dependence of the effects, I-V relationships of the peak tail current amplitudes before and after the administration of E-4031 and addition of EE2 were averaged (Figure 4B) and plotted versus the preceding test pulses from −40 mV to 60 mV. E-4031 reduced the peak hERG tail amplitudes measured after voltage steps to 20–60 mV, and the addition of EE2 at 1 nM partially recovered the reduced current amplitudes measured after voltage steps to 40–60 mV (Figure 4A,B). After normalization by the maximal peak amplitude recorded during the preceding voltage step at 60 mV in the control (Figure 4C), EE2-induced recovery from inhibition was observed in the tail amplitudes measured after voltage steps to −10 mV and 0 mV, where the hERG channel played a significant role in repolarization of the cardiac action potential. The tail current amplitude normalized to the maximum tail current amplitude at each I-V challenge was used to fit the activation curves shown in Figure 4D. No significant changes in midpoint voltage (V_50_) or slope factor (*k*) for activation were found by the application of drugs, whereas the decreased Imax by E-4031 significantly recovered after the addition of EE2 (Table 2), confirming the results shown in Figure 4.

## 4. Discussion

Synthetic estrogens, such as oral contraceptives, have medical applications worldwide [18]. Ethinylestradiol (EE2) is an estrogen medication used widely in oral contraceptives in combination with progestins [18]. In this study, EE2 reduced the hERG block by E-4031 without exhibiting detectable effects on the hERG channel function. As the hERG blockade by selective I_Kr_ inhibitors like E-4031 is associated with QT prolongation and an increased risk of arrhythmia, interaction with EE2 may reduce this risk. Because the risk of drug-induced QT prolongation is associated with physiological conditions such as heart rate [19] and genetic substrates such as LQT1 mutations [20], the clinical relevance of the degree of recovery of partial hERG block by EE2 may be case-by-case. The concentrations of EE2 used in this study (1–10 nM) are near or greater than peak concentrations in common clinical doses (1–5 nM) [21]. Thus, oral contraceptives containing EE2 may alter the risk of arrhythmia induction by a hERG inhibitor, E-4031.

We previously reported that 17-β-estradiol (E2), the most bio-active estrogen, promotes hERG inhibition and rodent QT interval prolongation caused by E-4031 [13,14]. As shown in Figure 1A, E2 alone partially inhibited the hERG channel described initially by Kurokawa et al. [13], which is different from the interaction with EE2 described in the present study. The partial inhibition of hERG channels by physiological serum concentrations of E2 [13] or estrone sulfate [15], but not by its precursors dihydrotestosterone or progesterone, was abolished when the aromatic side chain F656 located in the inner cavity of the hERG channel was mutated to threonine or methionine [13], suggesting that the aromatic ring in the chemical structure of E2 is the site of interaction. The interaction of E2 with hERG inhibition via the aromatic ring has also been demonstrated in vivo, as the QT-prolonging effects of E-4031 were increased by E2 in aromatase knockout mice lacking endogenous estrogen [14]. The effects of E2 on the risk of QT prolongation by hERG inhibition [14] suggest the general involvement of aromatic estrogens with or without independent inhibition of hERG [15], but other interactions between estrogens and hERG blockers have not been confirmed and require further investigation.

On the molecular level, we discuss the present results based on our previous MD simulations of the interaction between E2 and dofetilide [22]. The MD simulation using an hERG open state model identified a binding site for E2 which is defined by the aromatic F656 side-chain, supplemented by a number of hydrophobic residues including L650 and A653. Moreover, Y652 is also transiently involved at this site. The E2 intracellular cavity binding site is adjacent to that of dofetilide, suggesting a likely interaction. Because the chemical structure that binds it to the common binding site of hERG is almost identical for dofetilide and E-4031, a similar interaction between E2 and E-4031could be speculated. It is apparent that 17α-ethynylation of estradiol in EE2 alters the hydrophobic environment around side chains of Y652 and F656, implying that the effects of E2 and EE2 are so different. In the present study, we found that EE2 reduced the effects of E-4031 on hERG channels, as opposed to enhancing them, suggesting that estrogens can alter the action of hERG inhibitors in both directions. Whether EE2 was administered prior to (Figure 2) or after (Figure 3 and Figure 4) E-4031, EE2 similarly attenuated hERG inhibition. In addition, alanine substitution of Y652, a typical drug-binding site of the hERG channel, did not affect the action of E2 [13]. This suggests that the site of action of estrogens, including F656 of the hERG channel, is distinct from the typical binding site of hERG inhibitors, which includes both F656 and Y652. This is a reasonable hypothesis considering that hERG potassium channels exhibit numerous interactions with diverse chemical scaffolds [23]. Further studies on the binding site of estrogens in the hERG channel are expected in the future.

The clinical significance of our cell-based assay is that we confirmed that synthetic estrogens used in medicine can influence the action of hERG blockers. There is a worldwide debate concerning whether oral contraceptives increase the risk of arrhythmias caused by QT-prolonging drugs, and there are reports that some oral contraceptives increase the risk of TdP caused by *d,l*-sotalol [16]. In summary, this study suggests that the accuracy of the assessment of cardiotoxicity due to arrhythmogenesis caused by hERG inhibition can be improved by considering the interaction with estrogens.

## Figures and Tables

**Table 1:** Effects of EE2 on voltage-dependence of hERG activation. Before and after a cumulative 5-min exposure from 1 nM to 10 nM EE2, I-V relationships were obtained by the same voltage protocol described in Figure 1D–G. Eighteen cells were used. No significant difference was detected (ANOVA with repeated measures).

	Control (Before)	EE2 at 1 nM	EE2 at 10 nM
(*n* = 18)			
V_0.5_ (mV) Slope factor (*k*) *I*_max_ (pA/pF)	3.5 ± 1.5 10.3 ± 0.3 46.5 ± 3.5	4.4 ± 1.4 10.2 ± 0.3 47.3 ± 3.9	5.4 ± 1.3 10.5 ± 0.3 47.7 ± 3.9

**Table 2:** Effects of EE2 at 1 nM on the change in voltage dependence of the hERG activation by E-4031 at 30 nM. The experimental condition and results are also shown in Figure 4. Seven experiments were performed.

	Control	E-4031	E-4031 ± EE2
(*n* = 7)			
V_0.5_ (mV) Slope factor (*k*) *I*_max_ (pA/pF)	9.6 ± 3.2 8.1 ± 0.4 20.0 ± 7.7	11.1 ± 2.2 11.4 ± 0.9 9.2 ± 4.6 *	8.7 ± 5.3 10.9 ± 1.3 13.3 ± 5.7 ^#^

*p* < 0.05 ANOVA with repeated measures. * *p* < 0.05 Control vs. E-4031, ^#^ *p* < 0.05 E-4031 vs. E-4031 + EE2.

## Data Availability

Not applicable.
